# *HFE *H63D mutation frequency shows an increase in Turkish women with breast cancer

**DOI:** 10.1186/1471-2407-6-37

**Published:** 2006-02-19

**Authors:** Aysen Gunel-Ozcan, Sibel Alyılmaz-Bekmez, Emine Nilufer Guler, Dicle Guc

**Affiliations:** 1 Kirikkale University School of Medicine, Department of Medical Biology and Genetics, Kirikkale, Turkey; 2 Hacettepe University, Oncology Institute, Medical Oncology Department, Ankara, Turkey; 3 Hacettepe University, Oncology Institute, Basic Oncology Department, Ankara, Turkey

## Abstract

**Background:**

The hereditary hemochromatosis gene *HFE *plays a pivotal role in iron homeostasis. The association between cancer and HFE hetero- or homozygosity has previously been shown including hepatocellular and nonhepatocellular malignancies. This study was performed to compare frequencies of *HFE *C282Y and H63D variants in Turkish women with breast cancer and healthy controls.

**Methods:**

Archived DNA samples of Hacettepe University Oncology Institute were used in this study. The *HFE *gene was investigated by PCR-RFLP.

**Results:**

All subjects studied were free from C282Y mutation. Thirty-nine patients had H63D mutation and were all heterozygous. H63D allele frequency was 22.2% (39/176) in the breast cancer patients, and 14% (28/200) in the healthy volunteers. Statistical analysis of cases with *HFE *H63D phenotype showed significant difference between breast cancer and healthy volunteers (*P *= 0.02).

**Conclusion:**

Our results suggest that *HFE *H63D mutation frequencies were increased in the breast cancer patients in comparison to those in the general population. Also, odds ratios (odds ratio = 2.05) computed in this study suggest that H63D has a positive association with breast cancer.

## Background

The hereditary hemochromatosis gene *HFE *(6p21.3) 4 Mb telomeric to the *HLA-A *locus, and its product has a structure similar to MHC class I molecules [1]. It has a critical role in iron homeostasis. The total number of *HFE *variants detected to date is at least 37, of which 19 are missense [2]. Two mutations, C282Y (the substitution of tyrosine for cysteine at position 282) and H63D (the substitution of aspartate for histidine at position 63), are particularly frequent among patients with hereditary hemochromatosis [1] that is characterized by hepatic fibrosis and cirrhosis, diabetes mellitus, skin pigmentation, hypogonadism, arthropathy and cardiomyopathy as a result of excessive iron absorption from the gut and subsequent accumulation of iron in organs. HFE protein forms a stable complex with transferrin receptor (TFR), thereby reducing its affinity for transferrin (TF) by approximately ten fold [3]. TF is the major iron transport protein in blood and TFR facilitates the uptake of ironbound transferrin. C282Y mutation prevents the association of the mutant HFE protein with TFR because of the lack of cell surface expression [4]. As a result, increased affinity of the uncomplexed TFR for TF causes higher iron absorption. The significance of the H63D mutation on the *HFE *gene was at first controversial [1,3]. Subsequent studies suggested a functional role for H63D mutation [3,5,6]. The H63D variant of the HFE protein does reach the cell surface and forms a stable complex with TFR but fails to control high TFR affinity for TF [7].

*HFE *mutation frequencies show marked geographical differences throughout the world. Most Caucasian patients of Northwestern European ancestry with hereditary hemochromatosis are homozygous for C282Y [8,9]. Compound heterozygotes for the C282Y and H63D mutations expressing hemochromatosis are also observed, but with less penetrance than C282Y homozygotes [6,8]. Besides hepatocellular carcinoma [10,11] common malignancies, including hematological [12-14], colorectal cancer [15-17], and breast cancer [15,18] show risk associations with C282Y. However, there are contradictious studies showing no association between the *HFE *mutations and cancer risk [19-22].

One possible mechanism for the association might be the load of reactive free iron molecules which may cause DNA damage [23,24], which may suppress the host defense cells and induce proliferation [25,26], and which may convert nitric oxide from a proapoptotic to an antiapoptotic molecule [27].

The current report outlines our analysis of C282Y and H63D mutations in Turkish breast cancer patients and control subjects from the same geographical area.

## Methods

### Patients and Controls

In this study, we used 88 archived DNA samples, which had been isolated from blood samples of patients with breast cancer diagnosed at Hacettepe University Oncology Institute Department of Medical Oncology in Turkey over the last ten years. The mean age at diagnosis for the breast cancer patients was 41 yr (range, 21–75). The population controls consisted of 100 DNA samples from voluntary and healthy women who lived in Ankara, located at central Turkey. The mean age of the controls was 33 yr (range, 21–70). This study has been approved by the Hacettepe University Ethics Committee.

### PCR-RFLP

PCR amplification and electrophoresis were performed by using the standard procedures described previously [1,28]. The following general HFE primers were used at annealing temperature of 58°C and 57.4°C for analysis of C282Y and H63D mutations:

C282Y-Forward; 5'-TGGCAAGGGTAAACAGATCC-3',

C282Y-Reverse; 5'-CTCAGGCACTCCTCTCAACC-3',

H63D-Forward; 5'-ACATGGTTAAGGCCTGTTGC-3',

H63D-Reverse; 5'-GCCACATCTGGCTTGAAATT-3'.

**Figure 1:**
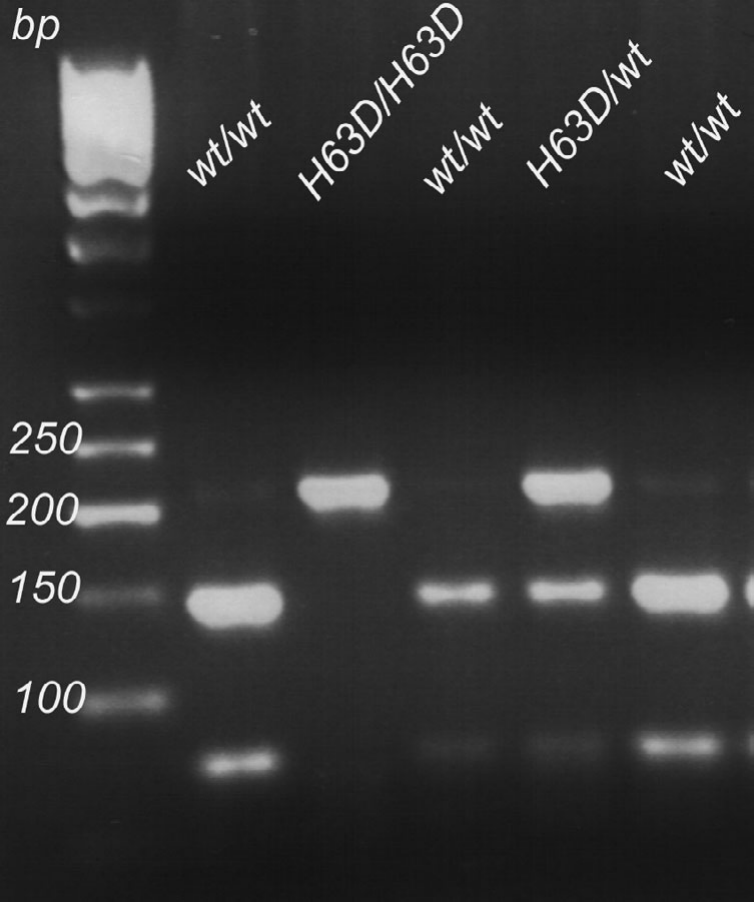
**Determination of H63D mutation of the *HFE *gene by restriction enzyme analysis**. Molecular weight marker (MW) sizes are shown on the first lane.

**Figure 2:**
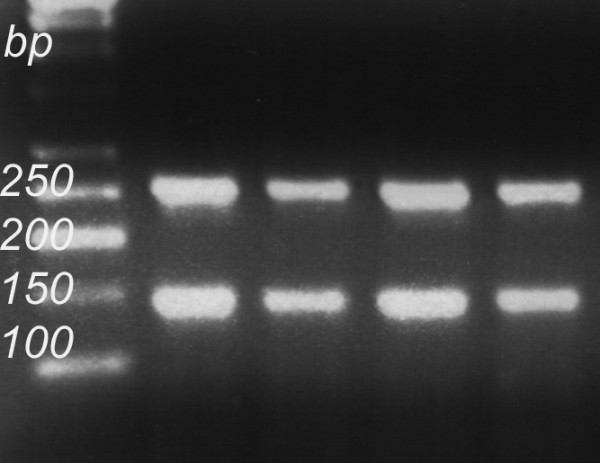
**Determination of C282Y mutation of the *HFE *gene by restriction enzyme analysis**. Neither homozygote nor heterozygote C282Y mutations were found in patients or in healthy controls.

The amplified fragments were digested with *Rsa*I for the C282Y and *Bcl*I for the H63D mutations. Upon digestion with *Rsa*I, the 387 bp PCR product of C282Y region shows two fragments of 247 bp and 140 bp in normal DNA and two additional fragments of 111 bp and 29 bp in mutant DNA. The 294 bp H63D region PCR product digested with *Bcl*I generates fragments of 138 bp, 70 bp in normal DNA and 208 bp in mutant DNA [28]. PCR digests were analyzed on 2.5% agarose gels (Figure 1 and 2). The relative positions of the mutations analyzed in the present study are given in Table 1.

### Statistical analyses

Frequencies of the *HFE *alleles were calculated by gene counting. Hardy-Weinberg equilibrium was tested before proceeding to the analysis. Statistical analysis of mutation prevalence in case and control groups was performed by using two-tailed Fisher's exact test. The proportions of patients with breast cancer and healthy volunteers who carried at least one mutant allele are presented with 95% confidence intervals (CIs) and Odds Ratio (OR). Analyses were performed by using Instat V2.02 (GraphPad Inc USA) statistical software package. In general group comparisons *P *value < 0.05 was accepted as statistically significant whereas in subgroup analysis after making Bonferroni adjustment *P *value < 0.0125 value was accepted as statistically significant.

## Results

According to the birth of place thirty-eight percent of the entire cases was from Central Turkey, twenty-eight percent was from South and West (Mediterranean region), twenty percent was from East, and fifteen percent was from North (Black Sea region). Information about cancer type and stages were available for 52 patients, and ninety-three percent of them had infiltrative ductal carcinoma and ninety-four percent of the women had limited-stage (stages I-III), 3 women (6%) had distant metastases at diagnosis.

There was no significant deviation from Hardy-Weinberg equilibrium in the distribution of genotypes in the control group. In the control group of 100 women, *HFE *H63D allele frequency was 14%, and the frequency of *HFE *H63D-homozygosity was 1%. Overall allele frequency for breast cancer group of 88 women (71 sporadic and 17 familial), for *HFE *H63D was 22.2% whereas there were no *HFE *H63D-homozygotes in the group. Statistical analysis of cases with *HFE *H63D phenotype showed significant difference between breast cancer and healthy volunteers (*P *= 0.02, OR = 2.05, 95% CI = 1.12 to 3.75) whereas there was no significant difference between either familial breast cancer and healthy volunteers or familial and sporadic breast cancer groups (Table 3). Subgroup analysis showed that the difference between the cases and control groups is based on the sporadic cases (P = 0.0001, OR = 3.13, 95% CI = 1.65 to 5.94). Information for the pre- and post-menopausal status was available for only 64 patients. Statistical analysis of cases with *HFE *H63D mutation among these patients showed no significant differences between premenopasual and postmenopausal breast cancer (Table 3). Characteristics of breast cancer patients with respect to *H63D *allele are summarized in Table 2. There were also no statistically significant differences between breast cancer patients with and without the H63D allele with respect to age or heredity. All subjects studied were free from C282Y mutation.

## Discussion

As expected from previous population studies [29-31] we did not find any C282Y mutation among the Turkish women who participated in this study. The results of this case-control study showed that H63D was more common in breast cancer patients. Because endogenous estrogens are more closely related to postmenopausal breast cancer and iron might have a role at redox-cycling estrogen metabolites to produce hydroxyl radicals [32,33], we compared premenopasual and postmenopausal breast cancer for the *HFE *H63D mutation. Since significant iron loss stops with menopause, an increase in *HFE*-associated cancer risk might also be possible. Contrary, there was no statistically significant difference between these groups (*P *= 0.12).

To the best of our knowledge, this is the second study that shows a significant association between female breast cancer and *HFE*-H63D although a subgroup analysis in a general cancer association study also revealed a non-significantly increased odds ratio for H63D carriers (n = 18, OR = 2.0, *P *= 0.14) in breast cancer [12]. A recent article, making the same comparisons of H63D mutation frequency between Russian women with breast cancer and controls, found age to be an important confounder of the association, wherein a positive association of H63D with breast cancer was only found among women over 57 years old [34]. Because of the insufficient number of the women over 45 years old in our study (Control N = 5, Cases N = 22), the estimation could be made in wide range which might cause unreliable interpretations. Therefore, age-specific analysis was not performed in this study. Another recent study showed no association between male breast cancer and H63D [35]. Although the lack of gender difference shown in association between *HFE *genotypes and medical conditions related to iron overload other than cancer [36], this variability in women with H63D mutations with respect to cancer risk could be the influence of potential modifier factors for breast cancer. The only other cancer association with H63D is with malignant gliomas [37]. If replicated elsewhere, further studies can be designed to distinguish between mechanistic possibilities. Our study had some limitations. We were unable to study the effect of H63D on serum iron parameters or gene and environment interactions, as we did not have stored serum samples from patients nor dietary or medication (oral contraceptives, etc) history. Ideally, a sample larger than ours should be studied in a genetic association study to rule out the chance factor. Not only is the study small, but when a small number of multiple comparisons are made, the power of the study is further diminished. When Bonferroni correction were made the comparison between the cases and controls in this study was no longer significant (*p *= 0.02 vs. *a *= 0.0125).

We compared the data on H63D frequencies from other population studies in Turkey. Our control frequency from age-matched females is in general agreement with other results which are 13.6%, 11.6% respectively [29,30]. The only exception is the study by Simsek et al., which reported a higher H63D mutation frequency (24.9%) from predominantly male blood donors [31]. A larger control group consisting of truly population-based females would have yielded a more robust estimate of the frequency if we had had the resources to achieve that.

The role of H63D in iron homeostasis is well established. Although an animal study unambiguously showed an effect of H63D mutation on hemochromatosis development [38], a human twin study established the influence of H63D on transferrin saturation levels [39]. Significant increases in both transferrin saturation and serum ferritin levels in H63D heterozygotes versus HFE wild type controls have also been demonstrated in large population studies [40-42]. *HFE *H63D mutation is very variable worldwide. The carrier frequency of the H63D mutation is 21.6% in Europe, 5.4% in Africa/Middle-East, 2.8% Asia and 22.8% in America [9]. One possibility is that H63D association with breast cancer might have been a selection of a specific subgroup among Turkish population. Turkey has a very heterogeneous population whose ancestries are from Anatolia (east Mediterranean region), Middle East and Central Asia. Related or unrelated susceptibility gene mutations, especially the ones which show ethnic dependence may both arise at higher frequencies in a specific subgroup of the mix-population. However, being a reference center Hacettepe Oncology Institute has the potential to comprise patients from different regions of Turkey and therefore, it is less likely that a specific subgroup of the population has been selected for this study.

Despite a large physical distance, the genetic distance between *HFE *and the telomeric end of the *HLA *complex is less than 1 cM. This results in linkage disequilibrium between H63D and *HLA*-A29 [43,44]. One alternative explanation is therefore confounding by locus, e.g., an *HLA *association may have appeared as H63D association in our study due to linkage disequilibrium. *HLA *loci have been studied in breast cancer and several associations have been reported but most importantly a recent large family study mapped a breast cancer susceptibility locus towards the telomeric end of the *HLA *complex [45]. The region between *HFE *and the *HLA *complex has another feature that may be relevant in the mechanism of an association with H63D. A significant correlation with CD8^+ ^T-cell numbers, but not with CD4^+ ^T-cell numbers, in subjects carrying both *HLA*-A29 and H63D or both *HLA*-A*01 and H63D mutation has been reported [44,46]. Thus, the H63D association may be a reflection of an immune response gene association in the region between *HFE *and *HLA*. Further large studies on *HFE *association with breast cancer susceptibility in populations lacking the C282Y mutation may need to include markers from *HLA *loci, loci between the *HLA *complex and *HFE *and other variants of the *HFE *gene for more meaningful results than our preliminary study has shown.

## Conclusion

We did not find any C282Y mutation among the Turkish women who participated in this study. The results of this case-control study show that H63D has a positive association with breast cancer. However, when a small number of multiple comparisons are made, the power of the study is diminished. Ideally, a sample larger than ours should be studied in a genetic association study to rule out the chance factor.

## Competing interests

The author(s) declare that they have no competing interests.

## Authors' contributions

AGO designed the study, carried out the molecular genetic (PCR-RFLP) studies, performed the statistical analysis and drafted the manuscript. SAB participated in the PCR-RFLP studies. ENG is the medical oncologist who diagnosed breast cancer patients and gave the related information. DG provided DNA samples of the patients and control group and helped to draft the manuscript. All authors read and approved the final manuscript.

## Pre-publication history

The pre-publication history for this paper can be accessed here:



## Figures and Tables

**Table 1:** HFE mutations with nucleotide changes and their positions within *HFE*

**Mutation**	**Nucleotide change**	**Position relative to initiating ATG **[GenBank: U60319]	**Position *in HFE***
C282Y	G → A	5473	Exon 4
H63D	C → G	3513	Exon 2

**Table 2:** Characteristics of breast cancer patients

Variable	*HFE *genotype	
	
	*H63D *allele^Ψ^	*Wt/Wt*^ξ^
Mean age at diagnosis in years (range)	42 (32–75)	40 (26–62)
Premenopausal at diagnosis	N = 22	N = 35
Familial	N = 8	N = 9
Early onset *	N = 12	N = 22
Sporadic	N = 31	N = 40

Ψ Heterozygous patients for H63D allele.

ξ *Wt/Wt*, homozygous wild-type.

* Patients diagnosed at younger than 40 years.

**Table 3:** Statistical analysis of cases with H63D mutation. Analysis between Total breast cancer patients and Healthy controls, Sporadic breast cancer patients and Healthy controls, Familial breast cancer patients and Healthy controls, Sporadic breast cancer and Familial breast cancer patients, Premenopausal and Postmenopausal breast cancer patients are shown. One healthy volunteer was homozygous for the mutation; all other subjects listed here were heterozygous. All the subjects studied were free from C282Y mutation.

	Cases with H63D mutation	*P *value (two-sided)	Odds ratio (OR)	%95 Confidence Interval-CI- (using the approximation of Woolf)
Total breast cancer patients: Healthy controls	39/88: 28/100	0.02	2.05	1.12 to 3.75
Sporadic breast cancer patients: Healthy controls	31/71: 28/100	0.05	2	1.05 to 3.78
Familial breast cancer patients: Healthy controls	8/17: 28/100	0.15	2.29	0.80 to 6.52
Sporadic breast cancer patients: Familial breast cancer patients	31/71: 8/17	1	1.15	0.4 to 3.32
Premenopausal breast cancer patients: Post menopausal breast cancer patients	22/57: 5/7	0.12	0.25	0.04 to 1.41
